# Sedentary Behavior and Physical Activity Associated with Psychosocial Outcomes in Adolescents with Type 1 Diabetes

**DOI:** 10.1155/2023/1395466

**Published:** 2023-04-05

**Authors:** Daniel R. Tilden, Amy E. Noser, Sarah S. Jaser

**Affiliations:** ^1^Endocrinology, Diabetes, and Clinical Pharmacology, University of Kansas Medical Center, Kansas City, KS, USA; ^2^Department of Family Medicine and Community Health, University of Minnesota Medical School, Minneapolis, MN, USA; ^3^Department of Pediatrics, Division of Pediatric Psychology, Vanderbilt University School of Medicine, Nashville, TN, USA

## Abstract

**Background:**

Adolescents with type 1 diabetes (T1D) are particularly vulnerable to poor psychosocial outcomes—high rates of diabetes distress and poor quality of life are common among this cohort. Previous work in the general population demonstrated positive associations between quality of life and increases in moderate-to-vigorous physical activity (MVPA), as well as decreased sedentary behavior. While survey-based assessments of young adults with T1D observed similar trends, these studies were limited by their use of subjective assessments of MVPA and sedentary behavior. The use of direct activity monitoring is needed to establish the association between psychosocial outcomes and MVPA and sedentary behavior among adolescents with T1D.

**Objective:**

To explore the association between objectively measured MVPA and sedentary behavior on psychosocial outcomes among adolescents with T1D. *Subjects and Methods.* The current study is a secondary analysis of baseline data collected for a pilot trial of sleep-promoting intervention for adolescents with T1D. Participants (*n* = 29, with a mean age of 15.9 ± 1.3 years) completed baseline surveys and wore an actigraph for a week following the baseline visit. We examined minutes per week of MVPA and proportion of awake time spent sedentary in relation to adolescents' diabetes distress, depressive symptoms, and diabetes-related quality of life.

**Results:**

Participants engaged in a mean of 19.6 ± 22.4 minutes of MVPA per day and spent 68.6 ± 9.9% of their awake time sedentary. MVPA was associated with lower diabetes distress in unadjusted (−3.6; 95% CI: −6.4 to −0.8) and adjusted (−2.6; 95% CI: −5.0–−0.3) analyses. Sedentary time was associated with higher diabetes distress in adjusted (6.3; 95% CI: 1.3–11.2) but not unadjusted (6.0; 95% CI: −5.6–12.6) analyses. In secondary analyses, we did not observe significant associations between quality of life or depressive symptoms with either MVPA or sedentary behavior. *Discussion.* Our findings extend previous survey-based work demonstrating an association between decreased diabetes distress with greater weekly MVPA and lower sedentary time. The current study highlights the multifaceted benefits of physical activity in this population and provides preliminary evidence for developing interventions to reduce sedentary time as an alternative method to improve psychosocial outcomes in this at-risk population.

## 1. Introduction

The International Society for Pediatric and Adolescent Diabetes and the American Diabetes Association recommend that children and adolescents with type 1 diabetes (T1D) engage in at least 60 minutes of moderate-to-vigorous physical activity (MVPA) per day [1–3]. These guidelines, similar to recommendations for youth without diabetes, are based on robust evidence of the impact of physical activity in reducing cardiovascular disease and improving glycemic control among youth with T1D [4–8]. While more established among adults with type 2 diabetes (T2D), recent work in youth with T1D has also demonstrated an association between lower levels of sedentary behavior and improved glycemic control [9–11]. Despite this evidence, most adolescents—with or without a chronic health condition—are sedentary most of the day and are not meeting recommendations for weekly physical activity [12–15].

Along with the cardiometabolic benefits, increased physical activity and reduced sedentary time are also associated with improvements in psychosocial outcomes in the general population [16–20]. Given the complexity of diabetes management during physical activity, understanding the impact of activity and reductions in sedentary time on psychosocial outcomes merits additional investigation. Emerging data among adults with T1D indicate improvements in psychosocial outcomes with increased physical activity and reductions in sedentary behavior [21–25]. Among adolescents with diabetes, a large, international study of adolescents ages 11–18 with T1D (*n* = 2,093) demonstrated that higher levels of self-reported MVPA were strongly associated with better quality of life, fewer psychological symptoms, and less worry [10]. In this same study, higher levels of sedentary behavior were also significantly associated with higher levels of worry and more psychological symptoms. However, a significant limitation of this previous work is the reliance on survey-based, self-reported measures of physical activity and sedentary behavior. These measures, while convenient, do not provide a reliable measure of adolescents' activity patterns (i.e., an assessment of the frequency, duration, and intensity of bodily movement) [26, 27].

In the current study, we evaluated the relationships between objectively measured physical activity and sedentary time and psychosocial outcomes including diabetes distress, depression, and diabetes-related quality of life among adolescents with T1D. Building on existing work using subjective activity measures, we hypothesized that higher levels of physical activity and lower levels of sedentary time would be associated with lower levels of diabetes distress as well as depressive symptoms and a better quality of life in this population.

## 2. Methods

Methods for participant recruitment and selection have previously been described [28]. To be eligible for the parent study, adolescents were between the ages of 13 and 17, had been diagnosed with T1D for >12 months, and reported insufficient sleep (<8 hours/night on most school nights). Adolescents and their caregivers provided written informed consent/assent in line with the protocol approved by the University Institutional Review Board (IRB#151271). The current analysis was performed using baseline data collected from participants at the time of study enrollment. After completing informed consent and assent, both the adolescent and parent completed psychosocial measures, and the adolescent wore an actigraph watch for one week.

### 2.1. Activity Measures

As a part of the parent study protocol, teens were instructed to wear actigraph monitors (Philips Respironics Spectrum Plus™) for seven nights. Activity counts were recorded at 60-second epochs and directly extracted from the days the monitor was worn. Sleeping and awake periods were determined using a composite score of activity and light data collected by the actigraph monitor. To ensure sufficient data for each participant and sufficient data to assess sedentary time, only participants with more than 48 hours of monitor use were included in analyses. Furthermore, similar to previous studies, participants with less than 10 hours of waking wear time per day, defined as periods with 0 accelerometer counts, were excluded to ensure adequate daytime observation of each participant [15, 29]. After data extraction, validated cut-points were applied to activity counts within each 60-second epoch to classify activity level as follows: <320 counts/min: sedentary; 1048–1623 counts/min: moderate physical activity; and ≥1624 counts/minute: vigorous physical activity [30–32]. Periods with either moderate or vigorous physical activity were summed and counted as MVPA. Sedentary time was defined as minutes with fewer than 100 activity counts per minute while awake.

### 2.2. Psychosocial Measures

Adolescent distress was measured using the Problem Areas in Diabetes-Teen Version (PAID-T), a measure of diabetes distress adapted for use in teens [33]. The PAID-T questionnaire consists of 26 items with scores ranging from 26 to 156 with higher numbers indicating higher diabetes distress [34]. Cronbach's alpha was 0.94.

Adolescents' diabetes-related quality of life was measured using the PedsQL 3.2 diabetes module, a 28-item questionnaire validated for use in children and adolescents with T1D [35]. Scores range from 0 to 100, with higher scores indicating increased quality of life. In the current sample, Cronbach's alpha was 0.81.

Adolescents reported depressive symptoms using the Patient Health Questionnaire (PHQ-9) [36]. This questionnaire assesses each of the 9 DSM-IV criteria for depression on a 0–3 scale, resulting in scores from 0 to 27. The PHQ-9 is widely used in adult and pediatric clinical care settings as well as among patients with T1D [37]. Higher scores indicate higher levels of depressive symptoms and have been associated with adverse glycemic outcomes among patients with T1D [38]. A score ≥10 is considered clinically significant. In the current sample, Cronbach's alpha was 0.73.

### 2.3. Glycemic Control

Hemoglobin A1C (HbA1c) is a measure of glycemic control over the prior 8 to 12 weeks. The point-of-care value from the clinic visit on the day of enrollment was extracted from participants' medical records.

### 2.4. Statistical Methods

Sedentary time and MVPA were the independent variables in the current analysis. Adolescent diabetes distress, as measured by the PAID-T, was the dependent variable for the primary analysis. Secondary outcomes were adolescents' quality of life (PedsQL) and depressive symptoms (PHQ-9). Bivariate correlations were conducted to identify associations between sedentary time, MVPA, and psychosocial factors. Multivariable regression analyses were conducted to adjust for covariates defined a priori, including participant age, sex, self-identified race, and baseline HbA1c. Each of these covariates was identified in the existing literature as potential confounders of the hypothesized relationship between MVPA, sedentary time, and psychosocial outcomes [39]. In particular, HbA1c has been shown to be associated both with physical activity and, separately, improvements in psychosocial outcomes [40–42]. All analyses were completed using Stata v16.1 (StataCorp, College Station, Texas).

## 3. Results

Among the larger cohort of participants included in the parent study, 29 participants had sufficient awake-time actigraphy data for inclusion in the current analyses. Participants' demographic information and summary statistics for activity and psychosocial outcomes are summarized in Table 1. The study participants were on average 15.9 years old ± 1.3 years with a mean HbA1c of 9.3% ± 1.9% at the time of study enrollment.

Compared to those excluded (*n* = 10) due to insufficient actigraphy data, participants included in the current analysis were more likely to be female (59% vs. 30%), but otherwise were similar in age (15.8 vs. 15.9 years) and had similar glycemic control (HbA1c 9.3% vs. 9.2%) (Supplementary Table 1).

On average, participants spent 68.6% ± 9.9% of their awake time sedentary, accumulating fewer than 100 accelerometer counts per minute during these periods, while spending an average of 19.6 ± 22.4 minutes per day in MVPA. Six participants (21%) met the ADA recommendation of 60 minutes of MVPA on at least one day of the study week with just 2 participants (7%) meeting the 60-minute goal on 5 days during the week of observation. Even when using the less stringent 30 minutes per day recommendation for adults, participants still frequently fell short of this recommendation with 10 participants (34%) never reaching it on any single day and 25 participants (86%) failing to meet the weekly activity goal.

In terms of psychosocial measures, the median PAID-T score was 61.3 (IQR 51.3–78.8) with 10/29 (35%) participants having PAID-T scores in the moderate distress range and one participant with a score indicative of high distress (3%). Pediatric quality of life scores had a mean of 30.2, while PHQ-9 scores had a mean of 3.37. Only 2 participants (7%) had PHQ-9 scores above the clinical cutoff (score of 10 or higher) for intervention. In our sample, age was not significantly associated with sedentary time or MVPA (see Table 2), and we did not observe any significant differences in sedentary time or activity level related to sex (*t* = 0.07, *p*=0.942 for sedentary time; *t* = −1.02, *p*=0.317 for MVPA).

In our primary analyses, sedentary time was associated with significantly higher levels of diabetes distress when adjusting for confounders, while our unadjusted analysis showed a similar, but nonsignificant trend (Table 3). Specifically, in our adjusted analysis, each percent increase of awake time spent sedentary correlated to nearly a half-point higher PAID-T score (0.48, 95% CI 0.04–0.93). This corresponds to an increase in distress of 3 points for each hour of increased sedentary time for an adolescent who is awake 16 hours per day. Similarly, both adjusted and unadjusted analyses showed a significant association between MVPA and lower levels of diabetes distress, with a 10-minute increment in MVPA correlating with a 2.6 point lower PAID-T score (95% CI: −5.0–−0.3) in our adjusted analysis (Table 4). Neither sedentary time nor MVPA was significantly associated with adolescents' depressive symptoms or quality of life in either adjusted or unadjusted secondary analyses.

## 4. Discussion

In this study, we sought to better understand the relationship between sedentary time, physical activity, and psychosocial outcomes in adolescents with T1D using objective measures of activity. Our primary analysis showed that higher levels of diabetes distress were significantly associated with higher levels of sedentary behavior and lower levels of MVPA. These findings are consistent with the previous literature among older people with T2D and with earlier studies in youth with T1D linking self-reported activity with quality-of-life measures [10, 43]. Our study extends these earlier findings by using accelerometry to directly measure participant activity and sedentary time. Given the limited nature of the activity data collected in the largest previous study (a total of five questions on activity including just one question measuring sedentary time), the current study is important in confirming these findings with more robust and reliable measures of adolescent activity [10]. Similar to studies using self-report, we found that the majority of adolescents were not meeting activity targets; only 2 participants met the daily goal of 60 minutes/day.

Given the frequently cited concern of patients, providers, and parents that physical activity and reduced sedentary time may result in increased rates of hypoglycemia, these findings have important clinical implications. While data from adults with T1D and the general population suggest that increased physical activity reduces distress, the complexity of blood sugar management before, during, and after exercise makes the nature of this relationship among adolescents with T1D less certain. For example, ISPAD guidelines recommend monitoring for hypoglycemia both during and up to 24 hrs after exercise with CGM [3]. Additionally, the influence of parental fear of hypoglycemia and differences in activity patterns during adolescence and adulthood, all make the nature of this relationship less certain for youth with T1D [44–46]. These data suggest that while hypoglycemia may be a complicating factor for physical activity among adolescents, the benefits of activity for both physical and psychosocial outcomes are, on net, more significant than these concerns.

In addition, our findings further extend our understanding of the relationship between sedentary time and psychosocial outcomes in this population. Previous work demonstrated the relationship between glycemic outcomes and sedentary behavior among those with T1D [11]. The current study suggests that this association between higher levels of sedentary time and adverse outcomes in this population may also extend to their psychosocial well-being. This association is supported by similar observations among adolescents and children without diabetes and provides further evidence to support the development of targeted interventions to reduce sedentary time alongside increasing physical activity in this population [17, 47, 48].

Despite these observed associations in our primary analysis, we did not find any significant associations between quality of life or depressive symptoms and MVPA or sedentary time. While the rates of elevated depressive symptoms in our sample were similar to those in previous studies, we did not observe significant relationships between these factors [49]. Likely owing to the limited power of our study, the modest associations between these factors were not observed in our sample. The negative findings in this study emphasize the need for further study and validation of our current findings.

This study had several strengths, including our use of an objective measure of physical activity through actigraphy and multiple measures of adolescents' well-being. The inclusion of multiple measures allowed us to distinguish between various potential benefits of exercise in this population (i.e., associations with depressive symptoms vs. diabetes distress). Limitations include the small sample size and a lack of racial/ethnic diversity or evaluation of other chronic stressors or other influences in the included sample. The majority (81%) of study participants were white and non-Hispanic, and while this is reflective of the clinic population, findings may not be generalizable to other populations. In addition, adolescents were only eligible for the parent study if they reported insufficient sleep (<8 hours/night), which is common among adolescents (>70%), but insufficient sleep has been associated with lower levels of self-reported physical activity and thus may have influenced our findings [50, 51]. It is also important to note that, as a sleep-focused study, the parent study did not require participants to wear their activity monitor during waking hours, and therefore, data for the current study were limited to participants who did not remove their accelerometer device. While unlikely, this may have selected a subset of patients distinct from the overall study population and resulted in a potential bias of these results.

## 5. Conclusion and Clinical Implications

The ADA and ISPAD guidelines both recognize the importance of physical activity and sedentary time for patients with T1D, but both only make specific recommendations for daily active time while they provide no recommendations for ideal durations of sedentary behavior [1]. Furthermore, the justification for these recommendations focuses mainly on the benefits to cardiovascular outcomes associated with physical activity. The current study highlights the psychological benefits of physical activity—particularly on diabetes distress—which may provide additional motivation to providers to emphasize physical activity as a key intervention in this population. Furthermore, our data demonstrating an association between sedentary time and diabetes distress suggest that even among adolescents for whom increased physical activity may not be feasible, reducing sedentary time alone may result in improvements in distress and overall well-being. Finally, given the rising recognition of the importance of psychosocial outcomes among patients with diabetes, future work further evaluating these relationships and the impact of interventions targeting physical activity or sedentary time should assess psychosocial outcomes—including diabetes distress—as potential benefits of these interventions [52].

## Figures and Tables

**Table 1:** Adolescent demographics and psychological outcome measures (*n* = 29).

Demographic factors	
Female sex (no., %)	17 (59%)
Race (no., %)	
White, non-Hispanic	22 (75.9%)
White, Hispanic	1 (3.5%)
Non-white	6 (20.7%)
Mean age (SD)	15.9 (1.3)
Mean BMI (SD)	25.0 (6.1)
Baseline mean HbA1c (SD)	9.2% (1.9)

Psychosocial measures (mean, SD)	
PAID-T	64.0 (17.7)
PedsQL diabetes module	72.9 (9.2)
PHQ-9	3.6 (3.3)

Activity measures (mean, SD)	
Average percent of daily awake hours spent sedentary	68.6% (9.9%)
Average minutes spent per day in MVPA	19.6 (22.4)
Days per week with more than 30 minutes of MVPA	1.4 (1.8)
Days per week with more than 60 minutes of MVPA	0.6 (1.4)

**Table 2:** The correlation matrix between psychosocial and activity measures and covariates.

	Percent sedentary time	Minutes/day in MVPA	PAID-T	PedsQL	PHQ-9	Age	Sex	Baseline HbA1c
Percent sedentary time	1							
Minutes/day in MVPA	−0.87^*∗∗*^	1						
PAID-T	0.34	−0.45^*∗*^	1					
PedsQL	0.25	−0.04	−0.33	1				
PHQ-9	0.15	−0.30	0.50^*∗∗*^	−0.37	1			
Age (years)	−0.06	−0.04	−0.05	0.12	0.27	1		
Sex	−0.04	0.28	−0.47^*∗*^	0.32	−0.22	0.06	1	
Baseline HbA1c	−0.06	−0.02	0.53^*∗∗*^	−0.33	0.07	−0.36	−0.15	1

^*∗*^Significant at the *p* ≤ 0.05 level. ^*∗∗*^Significant at the *p* ≤ 0.01 level.

**Table 3:** Models for outcome measures associated with differences in time spent sedentary.

	Change in psychosocial outcomes with 10 percent more sedentary time			
	Unadjusted model	*p* value	Adjusted model^*∗*^	*p* value
Difference in PAID-T	6.0 (95% CI: −5.6–12.6)	0.071	6.3 (95% CI: 1.3–11.2)	0.015
Difference in PedsQL	2.3 (95% CI: −1.2–5.8)	0.193	2.5 (95% CI: −6.9–5.8)	0.117
Difference in PHQ-9	0.5 (95% CI: −0.8–1.8)	0.443	0.5 (95% CI: −0.9–1.9)	0.453

^*∗*^Adjusted for sex, age, race, and baseline HbA1c.

**Table 4:** Models for outcome measures associated with differences in minutes/week of MVPA.

	Change in psychosocial outcomes with 10 minutes more weekly MVPA			
	Unadjusted model	*p* value	Adjusted model^*∗*^	*p* value
Difference in PAID-T	−3.6 (95% CI: −6.4–−0.8)	0.014	−2.6 (95% CI: −5.0–−0.3)	0.027
Difference in PedsQL	−0.2 (95% CI: −1.8–1.5)	0.842	−0.6 (95% CI: −2.1–1.0)	0.458
Difference in PHQ-9	−0.5 (95% CI: −1.0–0.1)	0.108	−0.3 (95% CI: −1.0–0.3)	0.257

^*∗*^Adjusted for sex, age, race, and baseline HbA1c.

## Data Availability

The data supporting the findings of the current study are available from the corresponding author upon request.

## Supplementary Materials

Supplementary Materials Supplemental Table 1: demographic summary statistics of participants in the parent study without sufficient actigraphy data for inclusion in the primary analysis.
