# Supplementary material for: Sedentary Behavior and Physical Activity Associated with Psychosocial Outcomes in Adolescents with Type 1 Diabetes
Source: Pediatr Diabetes. 2023 Apr 5;2023:1395466. doi: 10.1155/2023/1395466 (PMC10445792; doi:10.1155/2023/1395466)
Supplement: Supplementary Materials — Supplemental Table 1: demographic summary statistics of participants in the parent study without sufficient actigraphy data for inclusion in the primary analysis. [file 1395466.f1.docx]

**Supplementary Data**

Demographic summary statistics of participants in the parent study without sufficient actigraphy data for inclusion in the primary analysis.

**Supplemental Table 1**: Demographics of participants with insufficient actigraphy data

| **Demographics (n=10)** |  |
| --- | --- |
| Female Sex – no. (%) | 3 (30%) |
| Race |  |
| White, Non-Hispanic | 8 (80%) |
| White, Hispanic | 1 (10%) |
| Non-White | 1 (10%) |
| Mean Age (SD) | 15.8 (1.3) |
| Mean BMI | 24.6 (6.0) |
| Baseline HbA1c | 9.2% (1.8) |
| **Teen Psychosocial Measures (median, IQR)** |  |
| PAID-T | 50.0 (15.7) |
| PedsQL Diabetes Module | 74.4 (4.9) |
| PHQ-9 | 2.5 (3.7) |
